# Supplementary material for: DMRforPairs: identifying Differentially Methylated Regions between unique samples using array based methylation profiles
Source: BMC Bioinformatics. 2014 May 15;15:141. doi: 10.1186/1471-2105-15-141 (PMC4046028; doi:10.1186/1471-2105-15-141)
Supplement: Additional file 2 — DMRforPairs output for the comparison of A431-MCF7 and NA17018-NA17105. Please start from the HTML files in each folder. Available via the BMC Bioinformatics website. [file 1471-2105-15-141-S2.zip › 1394847754114233_MOESM2_ESM/A431_MCF7/figures/11269.pdf]

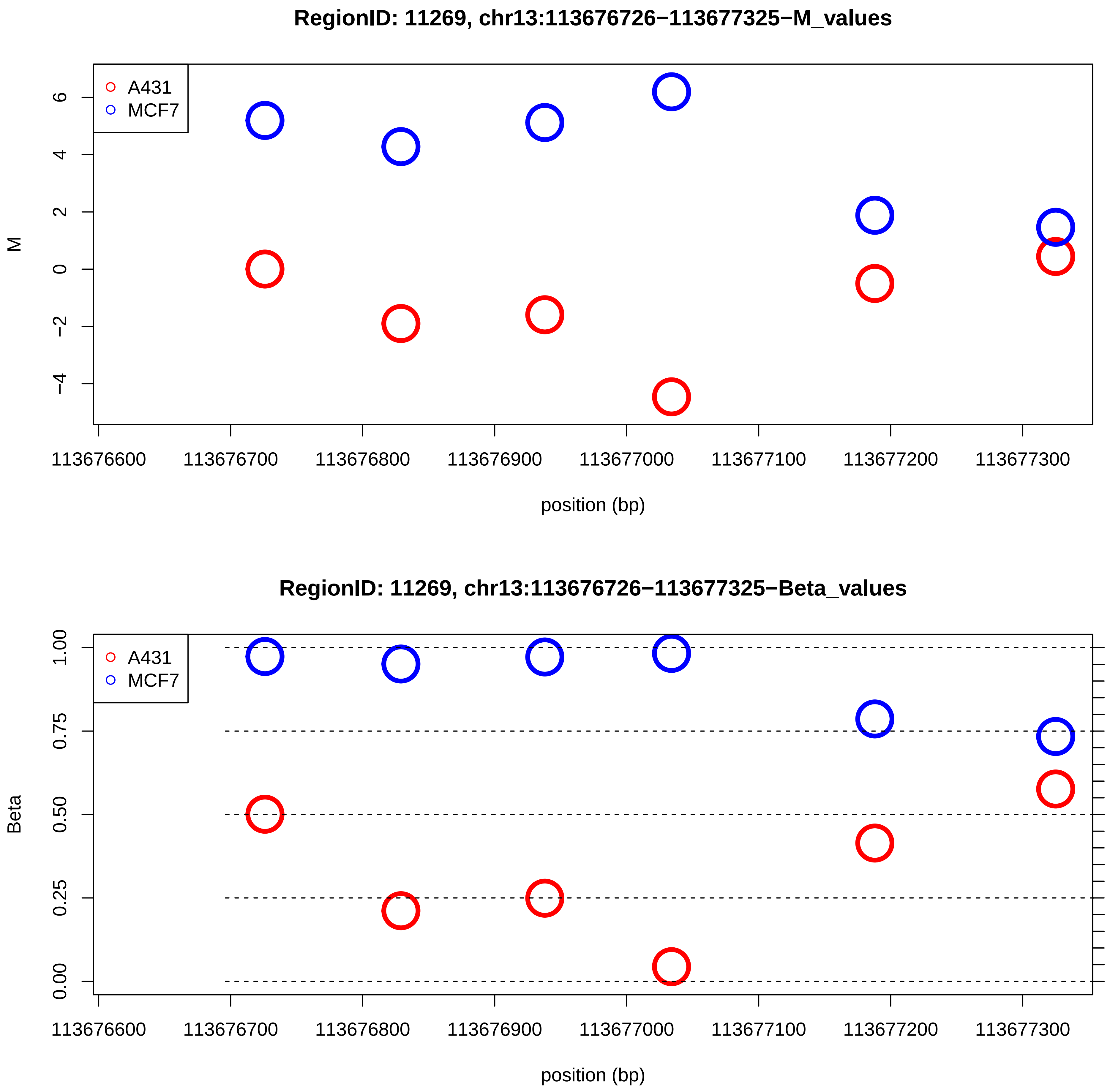
RegionID: 11269, chr13:113676726−113677325−M_values
A431
MCF7
M
6
4
2
0
−2
−4
113676600
113676700
113676800
113676900
113677000
113677100
113677200
113677300
position (bp)
RegionID: 11269, chr13:113676726−113677325−Beta_values
A431
MCF7
Beta
1.00
0.75
0.50
0.25
0.00
113676600
113676700
113676800
113676900
113677000
113677100
113677200
113677300
position (bp)

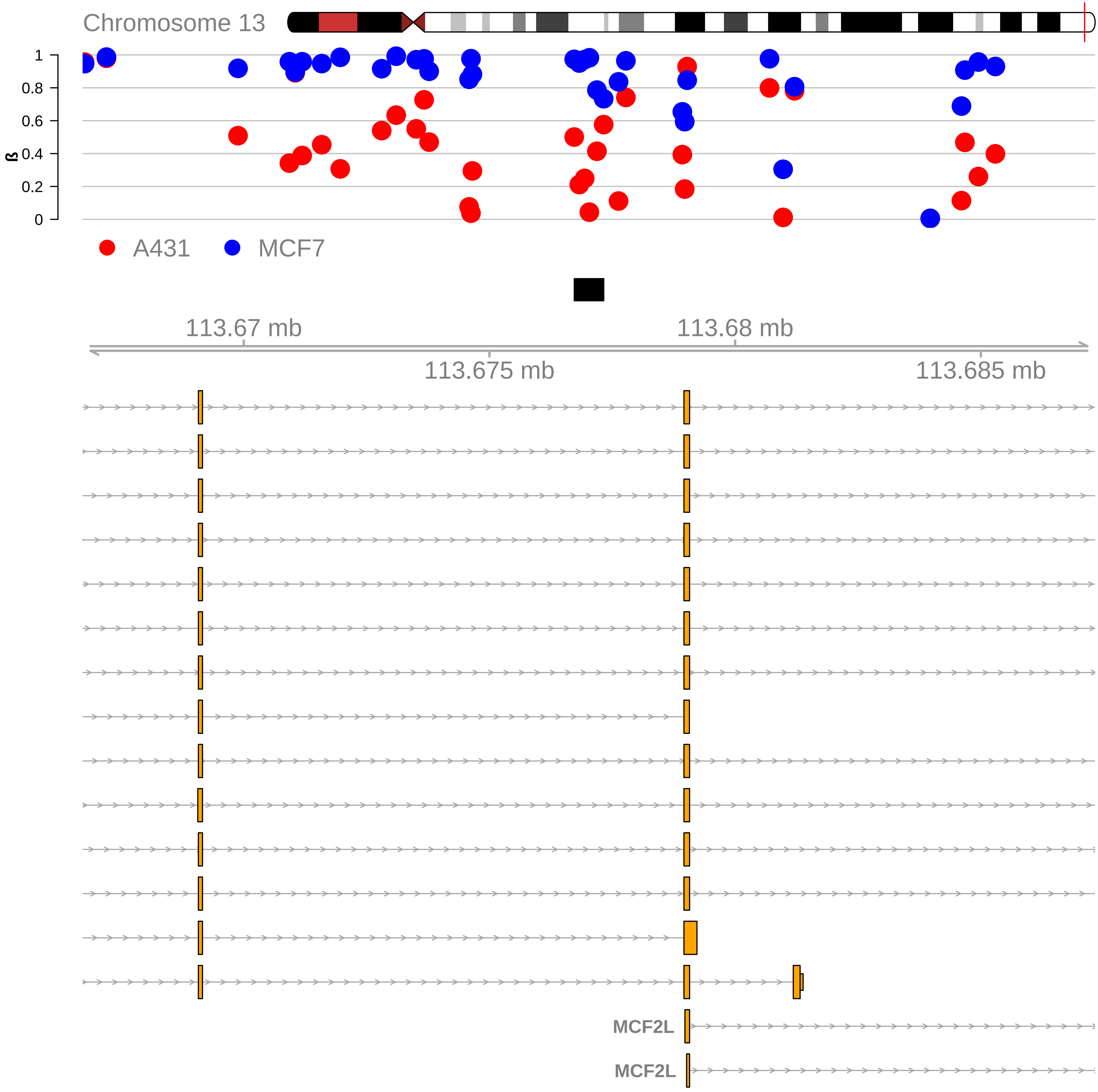

Chromosome 13
1
0.8
0.6
0.4
0.2
0
ß
A431
MCF7
113.67 mb
113.68 mb
113.675 mb
113.685 mb
MCF2L
MCF2L
